# Supplementary material for: Relationship between iron status markers and insulin resistance: an exploratory study in subjects with excess body weight
Source: PeerJ. 2020 Jul 31;8:e9528. doi: 10.7717/peerj.9528 (PMC7397981; doi:10.7717/peerj.9528)
Supplement: File S1 [file peerj-08-9528-s005.pdf]

**Organization's unique protocol**: AGL2014-55102-JIN_2

**Brief title**: Grape pomace polyphenols and cardiometabolic risk

**Official title**: Long-term effects of grape polyphenols as modulators of metabolic syndrome in humans.

**Acronym**: GRAPOM

**Study type**: interventional

**Record verification date**: February 2017

**Overall recruitment status**: Recruiting

**Study Start Date**: March 2017 (Anticipated)

**Primary Completion Date** (final data collection date for primary outcome measure): August 2017 (Anticipated)

**Study Completion Date**: March 2018 (Anticipated)

**Responsible Party**: Principal Investigator

**Investigator name**: Jara Pérez-Jiménez

**Investigator official title**: PhD

**Investigator affiliation**: National Research Council, Spain

**Sponsor** (Primary organization conducting study and associated data analysis (not necessarily a funding source): National Research Council, Spain

**Collaborators** (Organization(s) providing support: funding, design, implementation, data analysis or reporting. Required by International Committee of Medical Journal Editors (ICMJE) and World Health Organization (WHO). Enter **only the organization name**): Basque Country University, Spain.

**U.S. FDA-related drug**: No

**U.S. FDA-related drug**: No

**U.S. FDA IND/IDE Study**: No

- **Human Subjects Protection Review**:

  **Board Status**: submitted, approved

  **Approval number**:

  **Board name**: Clinical Research Ethics Committee

  **Board affiliation**: University Hospital Puerta de Hierro- Majadahonda

**Board contact**: secreceic.hpth@salud.madrid.org

**Data monitoring committee**: No

**Plan to share Individual Paticipants Data**: No

**FDA regulated intervention**: No

**Brief summary**: The aim of this is study is to evaluate the lomg-term effects of grape polyphenols in the modulation of markers of metabolic syndrome. Most of the previous works about polyphenols have only considered a fraction of polyphenols, i.e., extractable polyphenols. As a consequence, an important amount of dietary polyphenols, the so-called non-extractable polyphenols are ignored. In contrast, the effect of both extractable and non-extractable polyphenols will be considered in this study. Also, studies on the long-term effect of grape polyphenol on markers of metabolic syndrome have been mostly conducted in animals, so clinical trials on this topic are needed.

**Detailed description**: Fifty supposedly healthy volunteers with at least two cardiometabolic risk factors were recruited. The cardiometabolic risk factors used to select the subjects are described below.

The whole intervention will be divided in 2 periods, control (CTR) and dried and milled grape pomace (GRAPOM). The two periods will have a duration of 6 weeks. During the CTR period, subjects will follow their normal dietary habits and samples will be collected at the beginning and at the end. No proper placebo could be found for the product, so this CTR period was chosen. During the GRAPOM period, the subjects will daily consume 8 g of the product solved in water and samples will be collected at the beginning and at the end.

**Conditions or Focus of study**: subjects at cardiometabolic risk

**Keywords**: polyphenols, metabolic syndrome, cardiometabolic risk, grape pomace, postpandrial glucose, insulin resistance, obesity

**Intervention**: supplementation with grape pomace

**Study type**: interventional

**Primary purpose**: Basic Science

**Study phase**: N/A

**Interventional study model**: Crossover

**Model description**: GRAPOM: dried and milled grape pomaces. CTR: control period. All the subjects will pass the two periods: one half will do the CTRL period followed by the GRAPOM period, and the other half will do it inversely. The assignment to each order will be done randomly. Both period will be separated by a four weeks washing period.

**Number of arms**: 2

**Masking**: Investigator

**Allocation**: randomized

■ **Enrollment**:

**Number of subjects**: 50

**Type**: Anticipated

■ **Arm title**: GRAPOM

**Arm type**: Experimental

**Description**: Daily consumption for 6 weeks of 10 g of dried and milled grape pomaces solved in water. Samples will be collected at the beginning and the end of this period

■ **Arm title**: CTR

**Arm type**: No intrevention

**Description**: Follow-up for 6 weeks without intervention. Samples will be collected at the beginning and the end of this period

**Intervention type**: Dietary supplement

**Intervention name**: grape pomace (GRAPOM)

**Intervention description**: The whole intervention will be divided in 2 periods, control (CTR) and dried and milled grape pomace (GRAPOM). The two periods will have a duration of 6 weeks. During the CTR period, subjects will follow their normal dietary habits and samples will be collected at the beginning and at the end. No proper placebo could be found for the product, so this CTR period was chosen. During the GRAPOM period, the subjects will daily consume 8 g of the product solved in water and samples will be collected at the beginning and at the end.

■ **Primary outcome measure**:

**Title**: Fasting glucose and insulin

**Description**: Changes of blood glucose and insulin fasting levels as result of long-term supplementation with grape pomace (GRAPOM) , as measured by HOMA and QUICKI indexes

**Timeframe**: September 2017

■ **Secondary outcome measure (Outcome 2)**:

**Title**: Postprandial glucose and insulin

**Description**: Changes of blood glucose and insulin response after an oral glucose load as result of long-term supplementation with grape pomace (GRAPOM).

**Timeframe**: October 2017

■ **Secondary outcome measure (Outcome 3)**:

**Title**: Blood pressure

**Description**: Changes of blood pressure as result of long-term supplementation with grape pomace (GRAPOM).

**Timeframe**: October 2017

■ **Secondary outcome measure (Outcome 4)**:

**Title**: Blood cholesterol (total, HDL, LDL)

**Description**: Changes of blood cholesterol (total, HDL, LDL) as result of long-term supplementation with grape pomace (GRAPOM).

**Timeframe**: October 2017

■ **Secondary outcome measure (Outcome 5)**:

**Title**: Blood triglycerides

**Description**: Changes of blood triglycerides as result of long-term supplementation with grape pomace (GRAPOM).

**Timeframe**: October 2017

■ **Secondary outcome measure (Outcome 6)**:

**Title**: Blood C reactive protein

**Description**: Changes of blood C reactive protein as result of long-term supplementation with grape pomace (GRAPOM).

**Timeframe**: November 2017

■ **Secondary outcome measure (Outcome 7)**:

**Title**: Blood fibrinogen

**Description**: Changes of blood fibrinogen as result of long-term supplementation with grape pomace (GRAPOM).

**Timeframe**: November 2017

■ **Secondary outcome measure (Outcome 8)**:

**Title**: Blood uric acid

**Description**: Changes of blood uric acid as result of long-term supplementation with grape pomace (GRAPOM).

**Timeframe**: November 2017

■ **Secondary outcome measure (Outcome 9)**:

**Title**: Iron metabolism

**Description**: Changes of blood markers of iron metabolism as result of long-term supplementation with grape pomace (GRAPOM).

**Timeframe**: December 2017

■ **Secondary outcome measure (Outcome 10)**:

**Title**: Blood polyphenols

**Description**: Changes of blood polyphenols as result of long-term supplementation with grape pomace (GRAPOM).

**Timeframe**: December 2017

■ **Secondary outcome measure (Outcome 11)**:

**Title**: Urine uric acid

**Description**: Changes of urine uric acid as result of long-term supplementation with grape pomace (GRAPOM).

**Timeframe**: December 2017

■ **Secondary outcome measure (Outcome 12)**:

**Title**: Urine polyphenols

**Description**: Changes of urine polyphenols as result of long-term supplementation with grape pomace (GRAPOM).

**Timeframe**: January 2018

■ **Secondary outcome measure (Outcome 13)**:

**Title**: Body weight

**Description**: Changes of body weight as result of long-term supplementation with grape pomace (GRAPOM).

**Timeframe**: September 2017

■ **Secondary outcome measure (Outcome 14)**:

**Title**: Body fat

**Description**: Changes of body fat as result of long-term supplementation with grape pomace (GRAPOM).

**Timeframe**: September 2017

■ **Secondary outcome measure (Outcome 15)**:

**Title**: miRNA expression

**Description**: Changes of blood expression of selected miRNA as result of long-term supplementation with grape pomace (GRAPOM).

**Timeframe**: February 2018

■ **Secondary outcome measure (Outcome 16)**:

**Title**: Profile of fecal microbiota

**Description**: Changes of fecal microbiota as result of long-term supplementation with grape pomace (GRAPOM).

**Timeframe**: March 2018

**Sexes Eligible for Study**: All

**Gender based** (If applicable, indicate if participant eligibility is based on self-representation of gender identity): No

**Ages Eligible for Study**: 18 Years to 70 Years (Adult)

**Accepts Healthy Volunteers**: Yes

**Elegibility citeria**:

Inclusion criteria:

At least two of the following requirements:

- BMI ≥ 25 $kg/m^2$.
- Fasting glucose values ≥ 100 mg/dL.
- Triglycerides ≥ 150 mg/dL.
- HDL-cholesterol: ≤ 50 mg/dL women, ≤ 40 mg/dL men.
- Blood pressure: systolic ≥ 130 mm Hg or diastolic ≥ 85 mm Hg.

- Exclusion criteria:

- Subjects with a pharmaceutical treatment set to modify blood pressure, lipid profile or glucose.
- Volunteers participating in other studies or weight loss plans.
- Pregnant or breastfeeding women.

■ **Central contact person**:

**Name**: Jara
**Family name**: Pérez-Jiménez
**Title**: PhD
**Phone number**: +34 91 549 23 00
**Ext**: 231406
**Email**: jara.perez@ictan.csic.es

- **Facility**:

**Name**: Institute of Food Science, Technology and Nutrition (ICTAN-CSIC)
**City**: Madrid
**ZIP/Postal code**: 28040
**Country**: Spain

**Site recruitment status**: Recruiting

- **Facility contact**:

**Name**: Jara
**Family name**: Pérez-Jiménez
**Title**: PhD
**Phone number**: +34 91 549 23 00
**Ext**: 231406
**Email**: jara.perez@ictan.csic.es
