# Supplementary material for: Relationship between iron status markers and insulin resistance: an exploratory study in subjects with excess body weight
Source: PeerJ. 2020 Jul 31;8:e9528. doi: 10.7717/peerj.9528 (PMC7397981; doi:10.7717/peerj.9528)
Supplement: Supplementary file 5 [file peerj-08-9528-s004.docx]

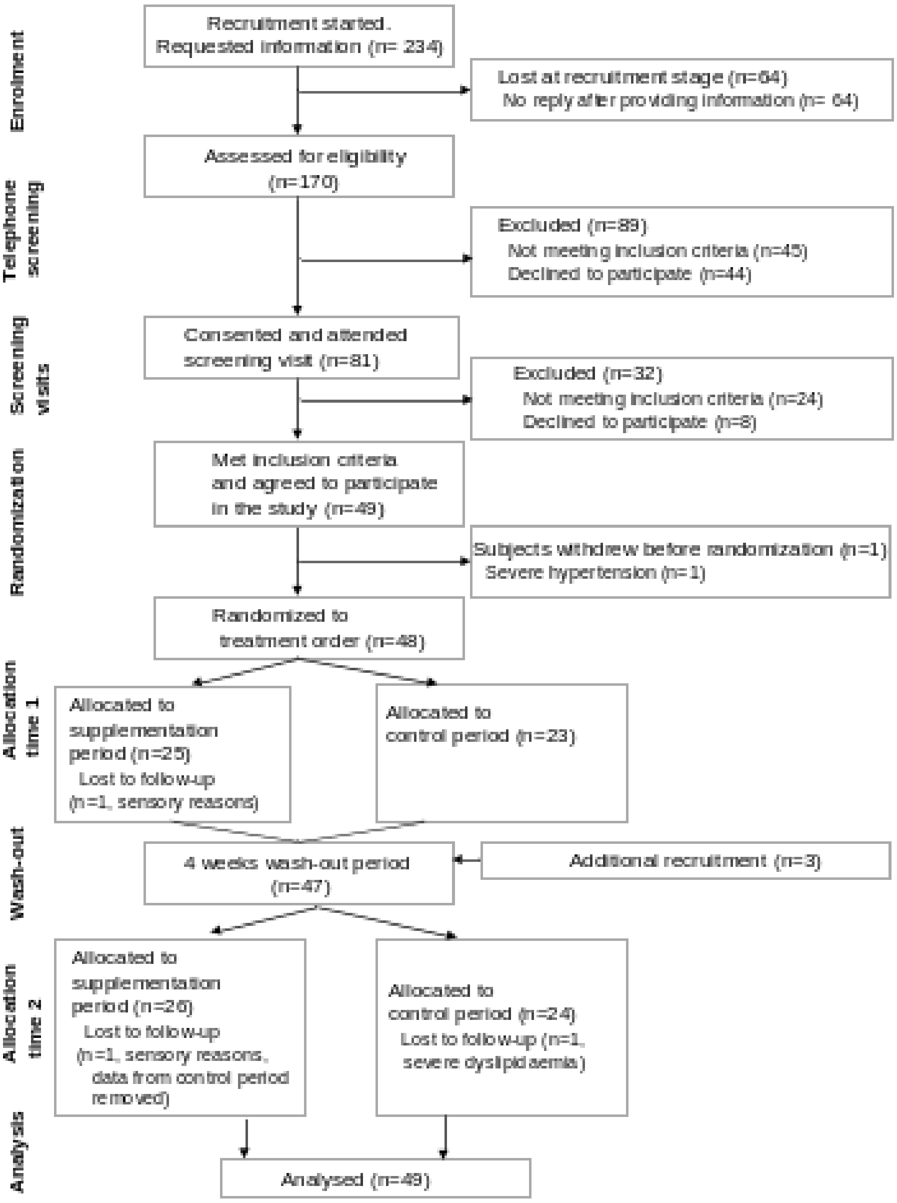
Enrolment
Recruitment started.
Requested information (n= 234)
Lost at recruitment stage (n=64)
No reply after providing information (n= 64)
Telephone screening
Assessed for eligibility
(n=170)
Excluded (n=89)
Not meeting inclusion criteria (n=45)
Declined to participate (n=44)
Screening visits
Consented and attended
screening visit (n=81)
Excluded (n=32)
Not meeting inclusion criteria (n=24)
Declined to participate (n=8)
Randomization
Met inclusion criteria
and agreed to participate
in the study (n=49)
Subjects withdrew before randomization (n=1)
Severe hypertension (n=1)
Randomized to
treatment order (n=48)
Allocation time 1
Allocated to
supplementation
period (n=25)
Lost to follow-up
(n=1, sensory reasons)
Allocated to
control period (n=23)
Wash-out
4 weeks wash-out period
(n=47)
Additional recruitment (n=3)
Allocation time 2
Allocated to
supplementation
period (n=26)
Lost to follow-up
(n=1, sensory reasons,
data from control period
removed)
Allocated to
control period (n=24)
Lost to follow-up (n=1,
severe dyslipidaemia)
Analysis
Analysed (n=49)
